# Functional Nanomedicines for Targeted Therapy of Bladder Cancer

**DOI:** 10.3389/fphar.2021.778973

**Published:** 2021-11-16

**Authors:** Chao Tang, Heng Liu, Yanpeng Fan, Jiahao He, Fuqiu Li, Jin Wang, Yuchuan Hou

**Affiliations:** ^1^ Department of Urology, the First Hospital of Jilin University, Changchun, China; ^2^ School of Chemical Engineering, Changchun University of Technology, Changchun, China; ^3^ Department of Dermatology, the Second Hospital of Jilin University, Changchun, China

**Keywords:** bladder cancer, targeted therapy, nanoparticle, intravesical instillation, chemotherapy

## Abstract

Bladder cancer is one of most common malignant urinary tract tumor types with high incidence worldwide. In general, transurethral resection of non-muscle-invasive bladder cancer followed by intravesical instillation of chemotherapy is the standard treatment approach to minimize recurrence and delay progression of bladder cancer. However, conventional intravesical chemotherapy lacks selectivity for tumor tissues and the concentration of drug is reduced with the excretion of urine, leading to frequent administration and heavy local irritation symptoms. While nanomedicines can overcome all the above shortcomings and adhere to the surface of bladder tumors for a long time, and continuously and efficiently release drugs to bladder cancers. The rapid advances in targeted therapy have led to significant improvements in drug efficacy and precision of targeted drug delivery to eradicate tumor cells, with reduced side-effects. This review summarizes the different available nano-systems of targeted drug delivery to bladder cancer tissues. The challenges and prospects of targeted therapy for bladder cancer are additionally discussed.

## 1 Introduction

Bladder cancer (BC) is the ninth most common urinary tract cancer type worldwide. In Western countries, the incidence of bladder cancer is different between genders, and the life time risk of bladder cancer is approximately 1.1 and 0.27% in men and women, respectively (Lenis et al., 2020). BC is reported to be the ninth leading cause of cancer-related mortality in men. In 2020, according to the American Cancer Society, 81,400 new BC cases and about 17,980 bladder cancer-related deaths were predicted in the United States alone (2020). In Europe, the incidence and mortality of BC are estimated as 27.1 and 8.9% respectively (Taskovska et al., 2020). Based on clinicopathological results, BC is classified into muscle-invasive bladder cancer (MIBC) and non-muscle-invasive bladder cancer (NMIBC, staged as Ta, T1, or carcinoma *in situ*) (Zhuo et al., 2016), the latter accounting for more than 75% cases (Babjuk et al., 2021). Transurethral resection procedures can effectively save the human bladder and enhance patient quality of life. However, studies have shown that without therapeutic drug interventions, the 5-years recurrence rate of NMIBC is ∼60–75% and about 15–25% of the recurrent tumor would progress into MIBC (Martinez et al., 2019; Seidl, 2020). Owing to its high recurrence rates, BC is a long-term disease that poses a heavy financial burden compared to other cancer types.

Thus, reducing the recurrence rate and preventing tumor progression remain significant challenges in the field of BC research. The gold standard treatment for superficial BC is complete transurethral resection and postoperative adjuvant intravesical instillation of chemotherapy to reduce recurrence and delay or even prevent progression to muscle-invasive disease. Clinical experience supports the utility of intravesical instillation therapy as an auxiliary method to prevent postoperative tumor recurrence. The drug contacts the bladder mucosa via this system to directly or indirectly kill the remaining tumor cells in the bladder, prevent tumor cell planting and reduce the recurrence rate.

Chemotherapy drugs and immune agents can be intravesically instilled into bladder directly via dissolving in saline. Chemotherapeutic agents for BC mainly include epirubicin (EPI), tetrahydropyranyl-adriamycin (THP) (Chen et al., 2012), gemcitabine (GEM) (Bohle et al., 2009), and mitomycin C (MMC) (Schmidt et al., 2020), which exert their effects through inhibiting tumor cell nucleic acid synthesis and influencing the cell cycle to induce cell death. *Bacillus* Calmette-Guérin (BCG) has long been approved as an immunotherapeutic agent (Guallar-Garrido and Julian, 2020) and its potential mechanism of action of BCG is that the absorption, internalization and subsequent induction of cytokines produced by BCG after intravesical instillation induce adaptive immunity and anti-tumor effects, thereby reducing the recurrence and progression of bladder cancer (Pettenati and Ingersoll, 2018; Han et al., 2020).

Clinically, intravesical drugs can reduce tumor recurrence rates to some extent. However, the treatment effect is significantly lower than expected, which may be attributed the following factors: 1) the drugs lack selectivity for tumor cells, leading to limitation of the tumor-killing effect, and exert significant toxic effects on normal bladder mucosa (Vasir and Labhasetwar, 2005); 2) due to the continuous production of urine, the concentration of drug within bladder cancer tissue is reduced, and with the continuous excretion of urine, the drug is excreted from the body, shortening its retention time in the bladder (GuhaSarkar et al., 2017); 3) drug efficacy is limited by the biological barrier of the bladder urothelium (Li et al., 2019); 4) owing to frequent administration and heavy local irritation symptoms, some patients cannot tolerate continual intravesical instillation. Therefore, the optimal strategy for therapy may be to enhance the ability of the drug to recognize bladder cancer tissue and extend its retention time on the target surface. Additionally, drugs could be efficiently internalized and taken up by tumor cells to achieve precise targeted cell killing, maximally inhibiting progression of tumor recurrence and reducing local side-effects.

Targeted therapy has received considerable research attention for further development. Targeted drug delivery systems present significant advantages in the treatment of bladder cancer by facilitating selective delivery of chemotherapy to tissues. However, achievement of therapeutic drug delivery specifically to tumor cells or tissues through the membrane and subsequent release of the drug into target tumors without damage to normal cells remains a significant challenge (Zheng et al., 2021c). Researchers have attempted to resolve this issue by using nanoparticles as delivery vehicles (Pridgen et al., 2007; Sarah C.; Andrew et al., 2012; Baetke et al., 2015). Numerous studies to date have developed nanotechnology with multiple features and functions for accomplishing efficient drug delivery and release.

Nanotechnology mainly deals with materials between 1 and 200 nm in size (Azocar et al., 2019). Since the concept of nanotechnology was first introduced in 1959 by Richard Feynman, rapid developments have occurred over several decades with considerable success in multiple fields, including chemistry, physics, biology and medicine (Bayda et al., 2019). In the field of medicine, nanotechnology has been successfully applied to diagnose and treat diseases using the properties (Zhang et al., 2020) and physical characteristics of nanomaterials at the molecular level (Betty et al., 2010). Nanomaterials have the characteristics of large specific surface area, strong adsorption capacity, high bioavailability, precise targeting characteristics and controlled release rate of drugs (Sibuyi et al., 2019). Nanotechnology has been used to effectively design and develop targeted vehicles providing drug delivery systems that transport therapeutic drugs into cancer cells through biological barriers. Relative to conventional drug therapy, nano-drugs have a higher surface area to volume ratio. Simultaneously, with advances in tunable optical, magnetic, electronic, and biological properties, nanomaterials can be developed with different sizes, shapes, chemical compositions, and surface chemical characteristics (Peer et al., 2007; Xia et al., 2009). Based on their multiple beneficial features, nanomaterials have significant potential as a new generation of drug delivery vehicles. To date, the FDA has approved 51 nanomedicines for clinical use and 77 products for clinical trials (Bobo et al., 2016).

**FIGURE 1:**
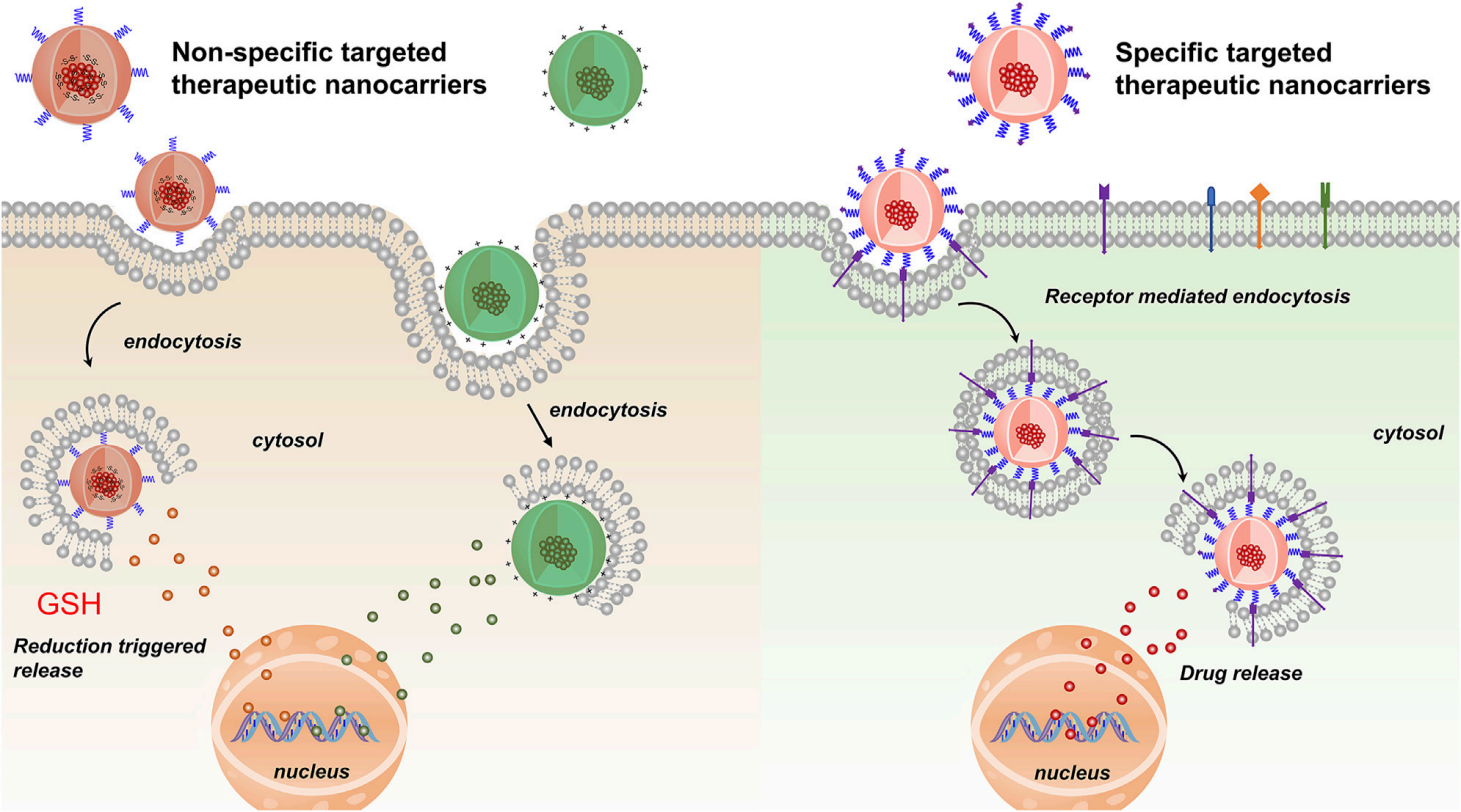
Non-specific and specific targeted therapeutic nanocarriers for treatment of BC.

Nanoparticles used as chemotherapy drug carriers are submicron-sized particles (3–200 nm), devices or systems generated using different materials including polymers, such as micelles, dendrimers, liposomes, viral nanoparticles, and even organometallic compounds (Cho et al., 2008). The nanomaterial surface is usually coated with a variety of polymers or specific biorecognition molecules for improved biocompatibility and targeting. For BC therapy systems, nanoparticles are divided into two types: non-specific and specific targeted therapy (Figure 1). The former type acts through interaction forces on the cell membrane surface to achieve specific targeted release of drugs, including surface charge, and besides, reduction-responsive nanoparticle is also one type of non-specific targeted nanoparticles. After being endocytosed into cells, under the action of high GSH, the disulfide bonds in reduction-responsive nanoparticles carrying therapeutic drugs are degraded, thereby releasing the therapeutic drugs and increasing the concentration of the drug in the cell, while the latter system binds cells through peptides or proteins and so on to achieve efficient cancer-targeted therapy (Table 1). The challenges and prospects of targeted therapy for BC are extensively discussed in this review.

**TABLE 1:** The mechanism of non-specific and specific targeted therapeutic nanocarriers for treatment of cancer.

Nanocarriers type	Nanocarriers properties	Material type	References
Non-specific targeted NPs	Positively NPs	CAT-TCPP/FCS	Li et al. (2020)
		CS-PCL	Bilensoy et al. (2009)
		(PLL−P (LP-co-LC)	(Guo et al., 2018; Guo et al. (2017)
	Reduction responsive NPs	P (LP-co-LC)	Guo et al. (2020b)
Specific targeted NPs	Small molecules-modified	FA-conjugated Au@TNA/MB	Hsu et al. (2020)
	Peptides-modified	Bld-1-KLA	Jung et al. (2016)
		MSNs@PDA-PEP	Wei et al. (2017)
		PLZ4-PNP	Lin et al. (2016)
		PLZ4-DNR/PTX	Pan et al. (2016)
		MPI/F-PEI	Wang et al. (2008)
		PCL-b-PEO	Zhou et al. (2013)
	Protein-modified	C225 antibody	Cho et al. (2019)
	Hyaluronic acid-modified	siRNA@CS-HAD	Liang et al. (2021)

## 2 Non-specific targeted therapeutic nanocarriers for treatment of Bladder Cancer

The efficacy of conventional instilled chemotherapy for bladder cancer is adversely affected by limitations in the ability of drugs to reach tumors (Arranja et al., 2017). During the treatment process, repeated instillation of drugs into the bladder is often necessary, which reduces the quality of life of BC patients and poses a serious burden to their families. To overcome the drawbacks of conventional chemotherapeutic agents, achievement of targeted effects on tumors is necessary. Recent studies have validated the potential of nanotechnology-based drug targeted delivery systems in achieving targeted drug release. The so-called targeted release of drugs refers to the delivery and release of chemotherapeutic drugs into target tumor sites, which should help reduce accumulation in non-tumor sites and improve their utilization and therapeutic effects (Moses. et al., 2003; Maeda et al., 2013; Bertrand et al., 2014).

Nanocarriers are commonly used to enhance the targeted release of drugs (Dadwal et al., 2018) but can encounter several obstacles in the process of drug delivery, such as the barrier effect of biological mucosa and non-targeted uptake of drugs. Therefore, nano-drug carrier system designs are generally based on the characteristics of the carrier to enhance targeted drug delivery. For example, a number of positively charged drug carriers (Choi et al., 2016; Young et al., 2016) and nanocarriers with reductive responsiveness (Hadi et al., 2021; Kuang et al., 2021; Oluwasanmi and Hoskins, 2021) are frequently used as non-specific drug delivery carriers.

### 2.1 Positive Nanoparticle Carriers for Bladder Cancer

Several types of nanoparticles have been investigated in clinical medicine applications including disease diagnosis and treatment. The carrier function of nanoparticles for drug delivery is a considerable focus of research. All nanoparticle applications are based on the uptake and utilization of nanomedicines by cells, with surface charge determined as an important factor affecting cellular uptake (Frohlich, 2012). According to differences in the nature of the surface charge, nanocarriers are divided into positively, neutrally and negatively charged nanoparticles. The BC cell surface has a large number of negative charges (Thomas et al., 2009; Marquez-Miranda et al., 2016). Two main ways for cells to take up positively charged nanoparticles have been identified, specifically, endocytosis (Hong et al., 2006; Xiang et al., 2012) and cell membrane perforation (Hong et al., 2006; Leroueil et al., 2007). Positive nanocarriers facilitate endocytosis of drugs by cells and overcome impermeability of cell membranes. For example, hydrophobic drugs are released from endosomes and move to targeted locations (Ramos et al., 2014). Positively charged nanoparticles thus have a higher affinity for cells relative to the other two nanoparticle types and enter cells more efficiently (Wu et al., 2016).

**FIGURE 2:**
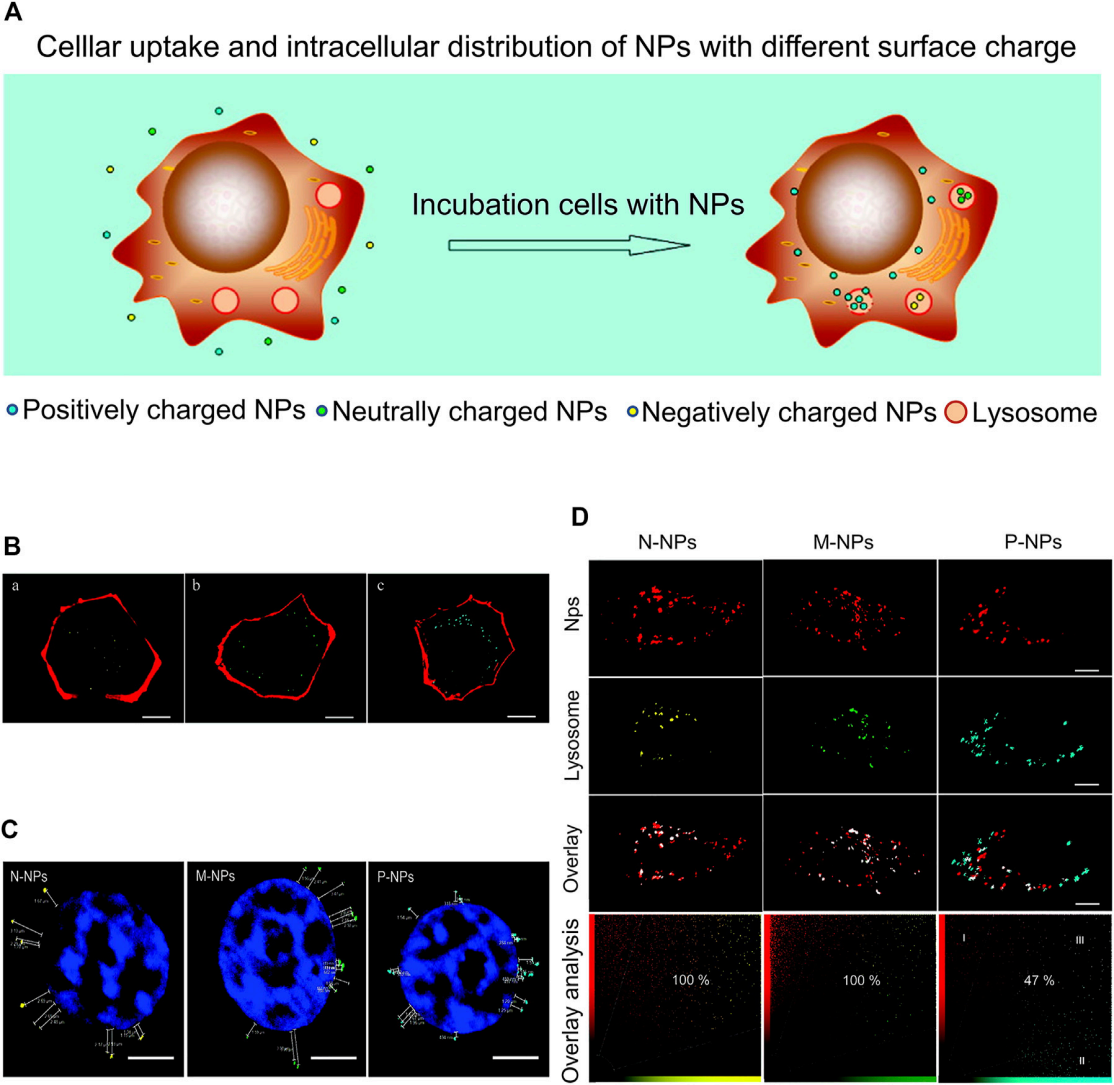
Surface charges affect cellular uptake. **(A)** Cellular uptake and intracellular distribution of NPs with different surface charges. **(B)** CLSM images of HKC cells incubated with NPs of different charges: **(A)** negatively charged, **(B)** neutrally charged, and **(C)** positively charged. **(C)** CLSM images of HKC cells after treatment with NPs of different charges. **(D)** CLSM images of lysosomes of HKC cells treated with different nanoparticles for 6 h. Reproduced with permission from (Yue et al., 2011).

Chitosan (polyglucosamine (1–4)-2-amino-BD glucose) is a product of deacetylation of chitin. Chitosan is the only polycationic polysaccharide in nature with unique biological properties amenable for clinical use, such as non-toxicity, good biocompatibility, and easy degradation (Zhang et al., 2016). This linear polyamine contains three active functional groups comprising hydroxyl groups that are easy to modify and graft. Based on its cationic properties (Guo et al., 2019; Guo et al., 2020a; Qiu et al., 2020), chitosan can be cross-linked with multivalent anions. Chitosan nanoparticles are one of the commonly used carriers for targeted delivery of anti-cancer drugs, including DNA or proteins that induce local genetic immunity. To exploit the unique beneficial properties of chitosan, different chemical modifications have been generated, providing a range of derivatives with properties useful for multiple applications (Yue et al., 2011). Based on chitosan modifications, several derivatives with distinct properties have been obtained to date, including o-carboxymethyl chitosan (Saranya et al., 2011), glycol chitosan (Koo et al., 2013), graft copolymerized chitosan (Prabaharan, 2015), and cross-linking chitosan derivatives (Amidi et al., 2010). In an earlier study, Yue and co-workers prepared three chitosan-based nanoparticles with distinct surface charges through SPG membrane emulsification and precipitation technology to assess surface charge effects on cellular uptake and intracellular transport. As shown in Figure 2, surface charge affected the uptake of cells and a positive charge could improve the rate of cell internalization.

The inner bladder surface has a biological barrier that prevents drug penetration. The bladder permeability barrier is caused by the thick mucus gel layer coating the bladder mucosa aligned with glycosaminoglycan chains dispersed in a viscous hydrogel (Erdogar et al., 2015).

**FIGURE 3:**
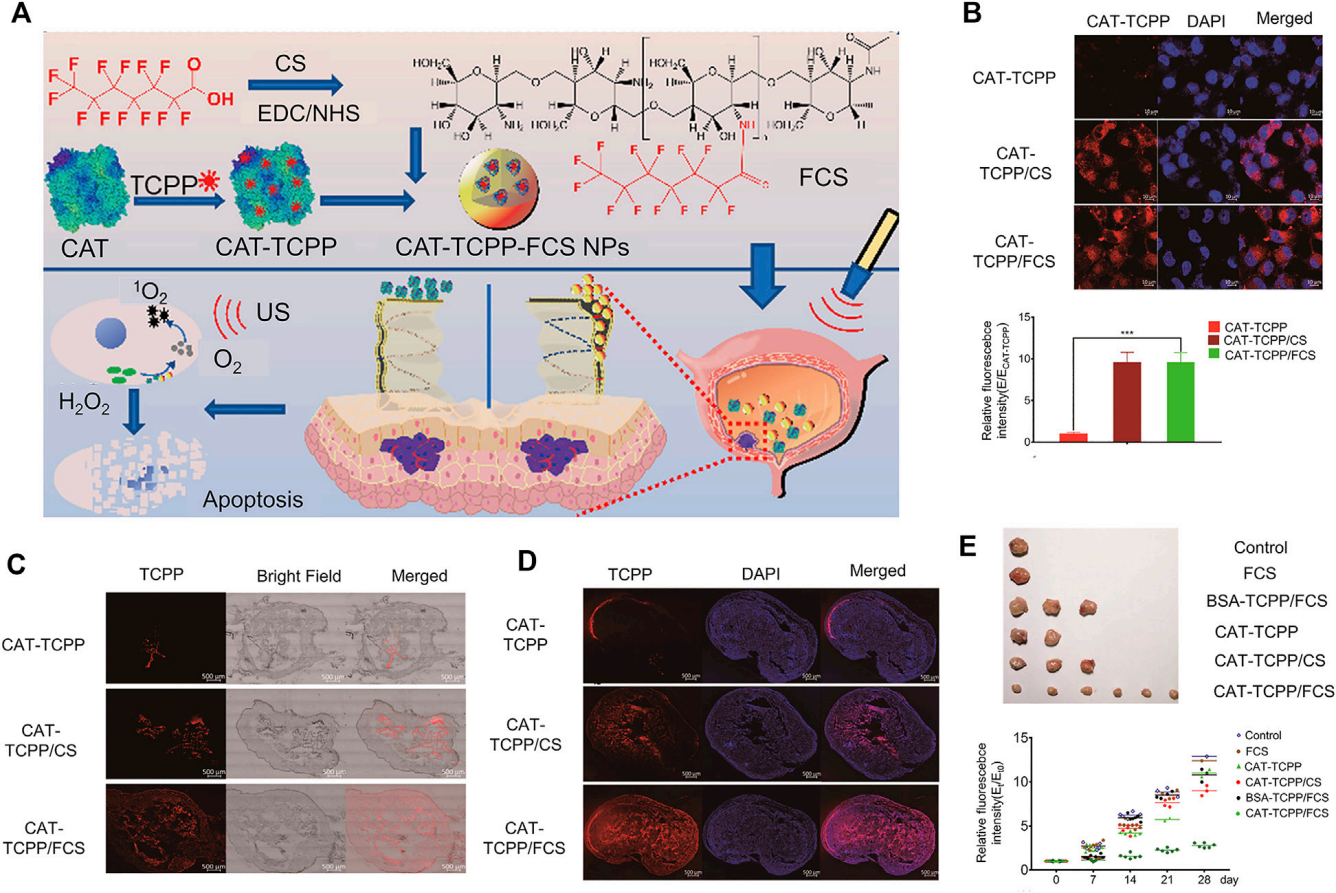
Fluorinated chitosan as a nano-delivery carrier of catalase for sonodynamic BC therapy post intravesical instillation. **(A)** Schematic diagram of preparation, characterization and intravesical instillation of CAT-TCPP/FCS NPs. **(B)** Confocal fluorescence images of MB49 cells treated with different nanoparticles. **(C)** Confocal fluorescence images of frozen sections of bladder treated with different nanoparticles for 12 h. **(D)** Confocal fluorescence images of mouse bladder cancer sections after intravesical instillation with different nanoparticles for 1 h. **(E)** Image of mouse bladder removed after the experiment and relative bioluminescence signal intensities following different treatments. Reproduced with permission from (Li et al., 2020).

Li and co-workers designed and synthesized a sonodynamic therapy (SDT) mucosal platform that autonomously generates transmucosal oxygen. In this system, fluorinated chitosan was used as an efficient and non-toxic transmucosal delivery carrier loaded with meso-tetra(4-carboxyphenyl) porphine-conjugated catalase. CAT-TCPP/FCS nanoparticles (NP) instilled into the bladder showed excellent transmucosal and tumor penetration ability. Simultaneously, CAT-TCPP/FCS nanoparticles effectively alleviated hypoxia in the tumor by catalyzing H_2_O_2_ in the targeted tumor sites to generate O_2_ through catalase and improved the therapeutic effect of SDT in ablation of bladder cancer under ultrasound (Li et al., 2020) (Figure 3A). Compared with bladder cancer cells treated with free CAT-TCPP, stronger CAT-TCPP fluorescence was observed in cells treated with CAT-TCPP/FCS nanoparticles, validating nanoparticle-enhanced cellular uptake (Figure 3B). As shown in Figures 3C–E, in the *in vivo* intravesical instillation experiment, bladder instilled with CAT-TCPP/FCS NPs showed stronger CAT-TPP fluorescence and deeper fluorescence penetration (Li et al., 2020). In this study, synthetic fluorinated chitosan was used as a protein carrier to achieve efficient transmucosal and intratumor delivery in the bladder. Fluorinated chitosan is considered a promising platform for intravesical instillation of drugs in addition to chitosan-based nanoparticles.

In a study by Bilensoy et al., positively charged poly-caprolactone coated with chitosan (CS-PCL) was used to load the intravesical chemotherapeutic drug mitomycin C (MMC). Notably, CS-PLC nanocarriers remained in the bladder for a long time, accumulated in bladder tumors, and prevented drug loss during urine excretion (Bilensoy et al., 2009).

In addition to positively charged chitosan-based nanoparticles, polyamino acid nanoparticles, such as poly-_L_-lysine (PLL), are commonly used as a positively charged drug delivery vehicle (Guo et al., 2017; Guo et al., 2018).

PLL is a water-soluble positive biopolymer that contains the monomeric lysine unit. Due to its inherent natural characteristics, such as non-antigenicity, biocompatibility and biodegradability, PLL is widely applied in multiple biomedical and pharmaceutical fields (Zheng M. et al., 2021). PLL has typical properties of polymeric nanoparticles. The amine group can be chemically conjugated with hydrophobic substances with carboxyl groups so that lipophilic drugs are effectively encapsulated during NP formation (Zhou et al., 2015). In addition to the advantages of conjugation, another important feature of the amino group of PLL is that its properties change under specific conditions. Specifically, in an acidic environment, the amino group is converted into a positively charged hydrophilic amino group that can interact with BC cell membranes through electrostatic interactions (Vasir and Labhasetwar, 2008). In a study by Guo et al., a cationic peptide nanogel (PLL−P (LP-co-LC) was designed to successfully deliver 10-hydroxycamptothecin (10-HCPT) (NP/HCPT) for intravesical instillation therapy to bladder cancer *in situ*. *In vitro*, NP/HCPT promoted endocytosis of drugs by cells and exerted obvious cytotoxicity, while *in vivo*, NP/HCPT displayed adhesion to bladder mucosa and strong penetration ability (Guo et al., 2017; Guo et al., 2018).

### 2.2 Reduction Responsive Nanoparticle Carriers for Bladder Cancer

**FIGURE 4:**
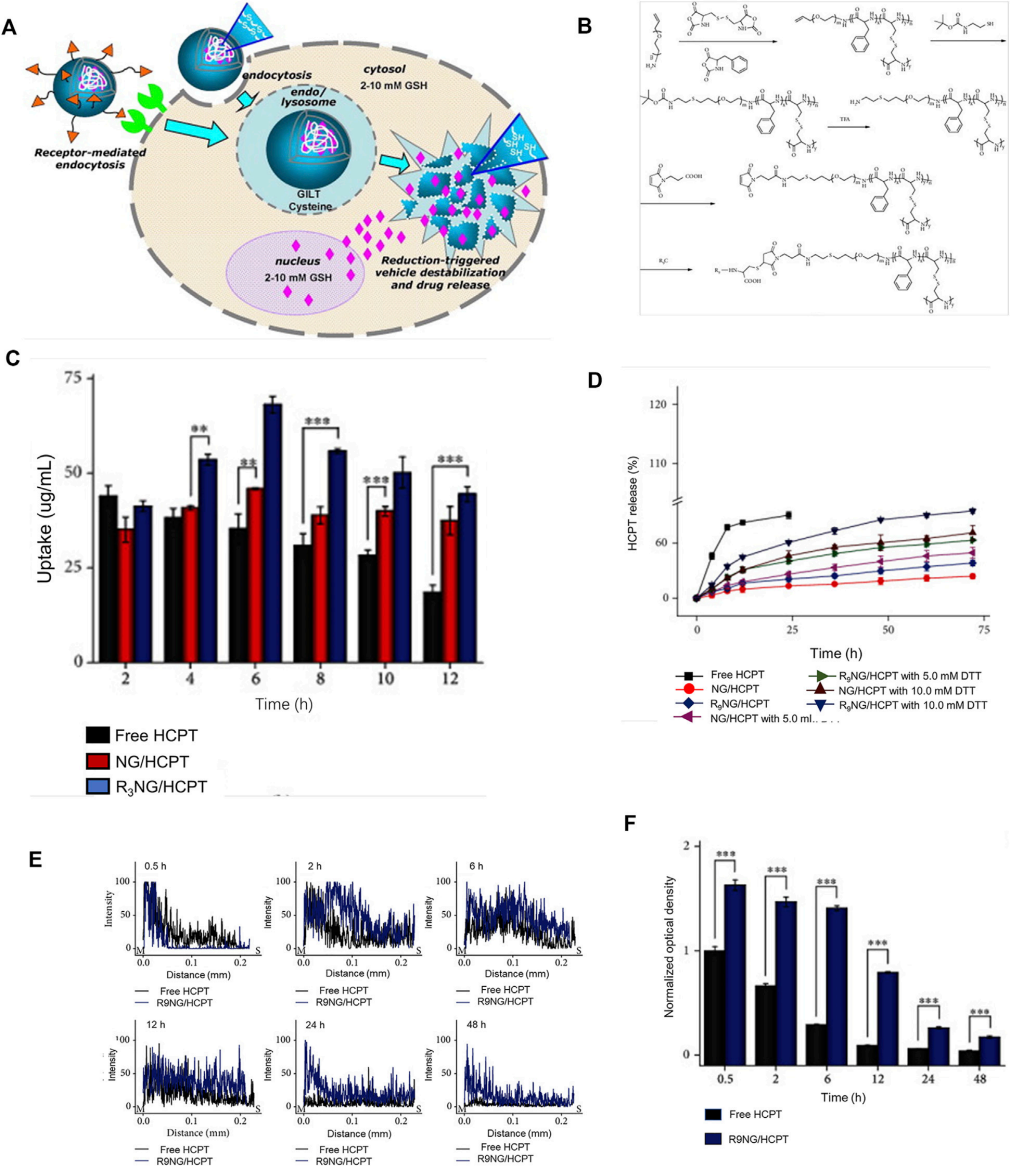
Reduced responsive nanoparticles as carriers for intracellular drug release. **(A)** Schematic illustration of reduced responsive nanoparticle carriers for intracellular drug release (Cheng et al., 2011). **(B)** Synthesis route of R9-PEG–P (LP-co-LC). **(C)** Intracellular HCPT release in human BC 5637 cells treated with different formulations of HCPT. **(D)** *In vitro* release profiles of R9NG/HCPT. **(E)** Quantification of bladder penetration of HCPT based on fluorescence intensity. **(F)** Quantitative analysis of fluorescence intensity of HCPT. Reproduced with permission from (Guo et al., 2020).

Several studies have reported that the intracellular environment of numerous tumor tissues is reductive. Under the same conditions, tumor tissues have high stability compared with normal tissues (Karimi et al., 2016), mainly due to the presence of a higher concentration of glutathione (GSH) (Asantewaa and Harris, 2021; Bansal and Simon, 2018). GSH is a common internal environmental stimulus in cells that rapidly destroys the stability of intracellular nanocarriers to achieve effective drug release (Cheng et al., 2011) (Figure 4A). Several researchers have focused on developing functional carriers with reduction responsiveness based on two strategies: insertion of disulfide bonds in the polymer backbone or using reduction-sensitive cross-linking molecules (Gulfam et al., 2017; Conte et al., 2018; Deng et al., 2018; Monteiro et al., 2020). Glutathione (GSH) levels inside and outside cancer cells are markedly different (Lai et al., 2021; Liu et al., 2021). Nanocarriers with reducing properties can achieve drug release more accurately and rapidly. After endocytosis of nanomedicine by cancer cells, under conditions of high GSH, disulfide bonds are reduced to a sulfhydryl group as a reduction response, causing the polymer to decompose and release the loaded drug (Zhao et al., 2017; Hu et al., 2018; Yang et al., 2021). With the rapid development of reduction-sensitive and reversible cross-linked polymers in the past few years, various reduction-sensitive systems have shown significant activity against different malignant tumors (Meng et al., 2009; Cheng et al., 2011; Feng et al., 2020). Bladder cancer cells contain a high concentration of GSH that can restore the microenvironment.

Guo and co-workers designed a disulfide bond cross-linked nanogel based on poly (L-phenylalanine-co-L-cystine) (Figure 4B). As shown in Figures 4C–F, the nanogel exerted obvious cytotoxicity in the human BC cell line 5,637. The longer residence time of the chemotherapy drug 10-HCPT in the bladder enhanced penetration ability into the bladder wall, leading to a significant inhibitory effect on tumors in animal models (Guo et al., 2020b).

## 3 Specific Targeted Therapeutic Nanocarriers for Bladder Cancer

Compared with the traditional method of administration, namely, direct intravesical drug instillation therapy, nanocarriers or biological materials exert greater effects on eliminating tumors and inhibiting tumor growth. However, ordinary nanomaterials have a number of drawbacks (Wei et al., 2021). The principle of action of nanocarriers is to extend the retention time of drugs and enhance penetration ability into bladder walls (Ding et al., 2019). However, no obvious advantages in active targeting of cancer cells have been observed, thus limiting the maximum therapeutic effect of drugs. Although a better curative effect could be achieved, a number of associated side-effects are reported. For treatment of BC, in addition to the use of nanocarriers with strong mucosal adhesion for delivery of drugs or passively targeted therapy, another potential strategy is active nanocarrier-mediated targeting of the tumor. This section introduces in detail nano-delivery vehicles that can specifically identify bladder cancer and reduce the toxicity of drugs to normal cells and tissues to enhance targeted treatment (Xu et al., 2020).

### 3.1 Small Molecule-Modified Targeted Therapeutic Nanocarriers for Bladder Cancer

Folic acid (FA, also known as vitamin B9) is a critical nutrient for growth and development of organisms that plays an indispensable role in DNA methylation, biosynthesis and repair. In nature, folic acid is widely distributed in various fruits and vegetables (Tu et al., 2018). Two mechanisms of FA uptake have been identified: 1) reduced folate carrier (RFC1) promotes endocytosis of folic acid in a reduced form by cells and 2) high-affinity folate receptor recognizes folic acid and promotes its oxidation by cells through receptor mediation. Folic acid that enters the cell through this pathway is released into the cytoplasm through endosomal acidification (Godeshala et al., 2016). FR has been shown to be overexpressed in a number of human tumor types, including triple-negative breast cancer, ovarian cancer, and non-small cell lung cancer. Additionally, the receptor is overexpressed in bladder cancer relative to normal bladder cells (Godeshala et al., 2016). The differential expression of FR between normal bladder and bladder cancer cells provides a significant research direction for targeted therapy.

**FIGURE 5:**
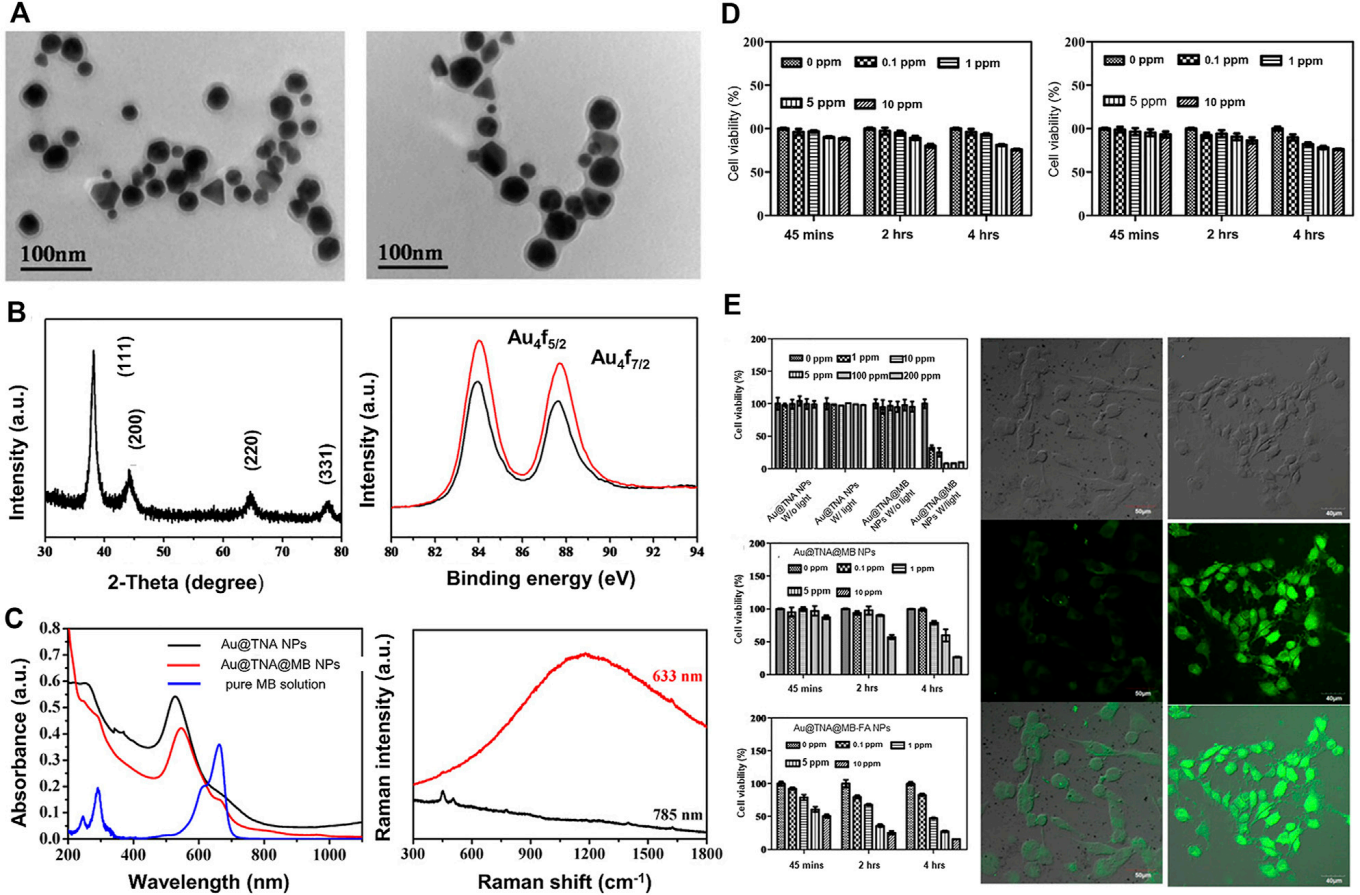
FA-conjugated Au@TNA@MB NPs for enhanced PDT therapy of BC cells. **(A)** Transmission electron microscopy (TEM) images of Au@TNA NPs and Au@TNA@MB NPs. **(B)** X-ray diffraction patterns of Au@TNA@MB NPs and X-ray photoelectron spectroscopy of two nanoparticle types. **(C)** UV-visible and fluorescence spectra of two types of NPs and Raman spectra of Au@TNA@MB NPs under different excitation wavelengths. **(D)** MTT assay at different time-points after treatment of SV-HUC-1 with two nanoparticle types. **(E)** MTT assay of T24 cells treated with nanoparticles under different conditions for 24 h. Photodynamic therapy of T24 cells treated with different nanoparticles stained with DCFH-DA dye. Reproduced with permission from (Hsu et al., 2020).

Based on the above findings, Hsu et al. designed and prepared Au@TNA core-shell nanoparticles via tannic acid (TNA)-assisted reduction of HAuCl_4_. Next, the photosensitizer methylene blue (MB) was combined on the surface of Au@TNA to generate Au @TNA@MB nanoparticles. Subsequently, nanocarriers were modified with folic acid (FA) to obtain FA-Au@TNA/MB NPs. The newly generated nanocarrier was composed of two types of nanoparticles (spherical and triangular) with an average size of 26.43 ± 3.7 nm (Figures 5A–C). *In vitro*, the photosensitizer exerted slight toxicity on the normal urothelial cell line SV-HUC-1 (Figure 5D) but aggregated significantly in the bladder cancer cell line T24, leading to reduced survival of cancer cells (Figure 5E). Therefore, FA-conjugated Au@TNA/MB NPs present a promising PS nanomaterial for treatment of NMIBC. Further animal experiments are required to confirm the therapeutic effects and adverse effects of these nanomaterials on bladder cancer (Hsu et al., 2020).

### 3.2 Peptide-Modified Specific Targeted Therapeutic Nanocarriers for Bladder Cancer

Compared with other targeted therapy, peptide-modified nanocarriers offer several advantages including lower production costs, relative stability, effortless commercialization, simple operation, and reduction of the immune response. At the same time, binding of peptides to receptors can achieve precise targeted therapy through design of specific reactive groups. In addition, particle size is relatively low, which has little effect on the physical and chemical properties of the synthesized nanoparticles (Zhong et al., 2014).

#### 3.2.1 Bld-1-Modified Specific Targeted Therapeutic Nanocarriers for Bladder Cancer

Recent studies have focused on screening of specific organs. The majority of tumor-specific peptides have been identified from phage display libraries. As many as 10^10^ known peptides exist in the phage library (Koivunen et al., 1999; Smith, 1985; H. MICHAEL; Ellerby et al., 1999; ohanna A.; Joyce, 2003). One notable example is the RGD motif comprising three amino acids, which binds tumor vascular endothelial cells through αvβ3 integrin (expressed specifically in vascular endothelial cells). Compared with their normal counterparts, tumor cells display upregulation of a number of growth factor receptors that can serve as molecular markers. For instance, epidermal growth factor receptor, urinary glycoprotein and α6β4 integrin are highly expressed in bladder cancer.

Lee et al. used a phage display peptide library to identify peptides specifically targeting BC cells. Several peptide sequences with a common motif, CXNXDXRX/RC, were selected. As discussed above, the inner wall of the bladder has a special protective layer composed of glycosaminoglycans and mucin. Although this layer can protect urothelium from adhesion of chemicals and microorganisms in concentrated urine, it also hinders intravesical instillation therapy. Effective penetration of the drug at this time shortens the action time. One of the peptides screened (CSNRDARRC) showed strong selective binding to bladder cancer cells. Fluorescent labeling experiments further revealed that CSNRDARRC co-localized with cytokeratin in bladder cancer tissues. Importantly, the peptide was not detected in normal bladder tissues and other organs. The tumor cell selectivity of CSNRDARRC and its specific targeting of bladder cancer strongly support the utility of this peptide as a BC-targeted therapeutic agent (Lee et al., 2007).

Jung et al. utilized the CSNRDARRC peptide (Bld-1) as a targeting ligand to selectively deliver KLA to bladder cancer and examined the activity of Bld-1-KLA hybrid peptide as targeted therapy for bladder tumors with pro-apoptotic peptides. The hybrid peptide effectively targeted bladder cancer cells and induced apoptosis, and could therefore serve as an excellent bladder tumor-targeting pro-apoptotic peptide (Jung et al., 2016).

**FIGURE 6:**
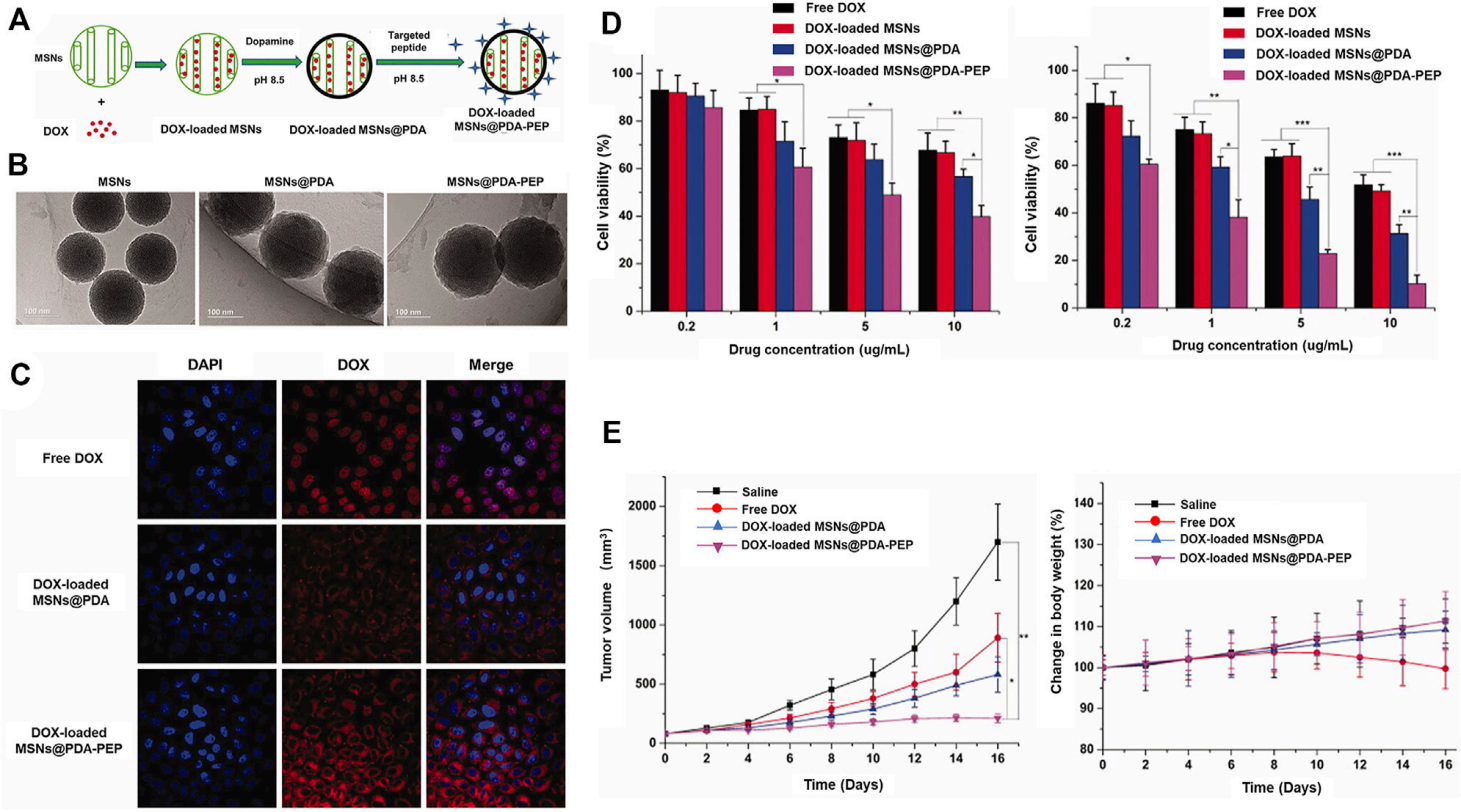
Utility of MSNs@PDA-PEP as a drug carrier for targeted BC therapy. **(A)** Schematic representation of the preparation techniques of DOX-loaded MSNs@PDA-PEP. **(B)** TEM of different NPs. **(C)** CLSM imaging of HT-1376 cells treated with different drugs for 2 h. **(D)** Viability of HT-1376 cells treated with different formulations of drugs after 24 and 48 h. **(E)** Tumor growth curves and weight changes of tumor-bearing nude mice treated with different formulations of drugs. Reproduced with permission from (Wei et al., 2017).

In addition, the Bld-1 peptide itself may be applied as a targeting ligand in combination with nanocarriers loaded with conventional chemotherapeutics for drug delivery to bladder tumors. Wei et al. (Wei et al., 2017) described a simple surface modification method based on polydopamine (PDA). The group prepared a novel mesoporous silica as a doxorubicin-loaded nanocarrier and used peptide (CSNRDARRC)-coupled targeted delivery for bladder cancer nanotherapy. The synthesis process is shown in Figure 6A. Further characterization of the synthesized drug-loaded nanoparticles revealed a nanoparticle size of 168.3 ± 8.1 nm for DOX-loaded MSNs@PDA and 170.2 ± 7.5 nm for DOX-loaded MSN@PDA-PEP, respectively (Figure 6B). Cell experiments were developed to verify endocytosis of the peptide-modified drug-loaded DOX-loaded MSN@PDA-PEP and its killing effect (Figures 6C,D). As shown in Figure 6E, DOX-loaded MSN@PDA-PEP significantly inhibited tumor growth in animal experiments. In conclusion, DOX-loaded MSN@PDA-PEP could specifically recognize BC cells, increase drug toxicity and suppress growth of BC.

#### 3.2.2 PLZ4-Modified Specific Targeted Therapeutic Nanocarriers for Bladder Cancer

The phage display library was screened for peptides that could be used in bladder cancer therapy (Lee et al., 2007). *In vivo* targeting of human primary bladder cancer cells was a subsequent focus of a study by Zhang et al. A one-bead one-compound combinatorial peptide library technology (OBOC) was applied to identify several bladder-specific ligands with the same DGR motif showing potential utility in targeted bladder cancer therapy. However, only the cyclic PLZ4 peptide with the amino acid sequence cQDGRMGFc was specific for bladder cancer. PLZ4 could specifically bind αvβ3 integrin on BC cells but not normal urothelial cells (Zhang et al., 2012).

Lin and co-workers developed a multifunctional nanoporphyrin platform modified with PLZ4 ligand. PLZ4-nanoporphyrin (PNP) has multiple applications in photodynamic diagnosis, photothermal therapy and targeted chemotherapy. PNP is a nanosphere that emits fluorescence/heat/reactive oxygen species upon irradiation with near-infrared. Compared with free Dox, the PLZ4-PNP platform significantly prolonged overall survival of mice. The three effective modes (photodynamic/photothermal/chemistry) of the platform could be easily applied for individualized treatment and have significant potential in the targeted management of bladder cancer (Lin et al., 2016).

**FIGURE 7:**
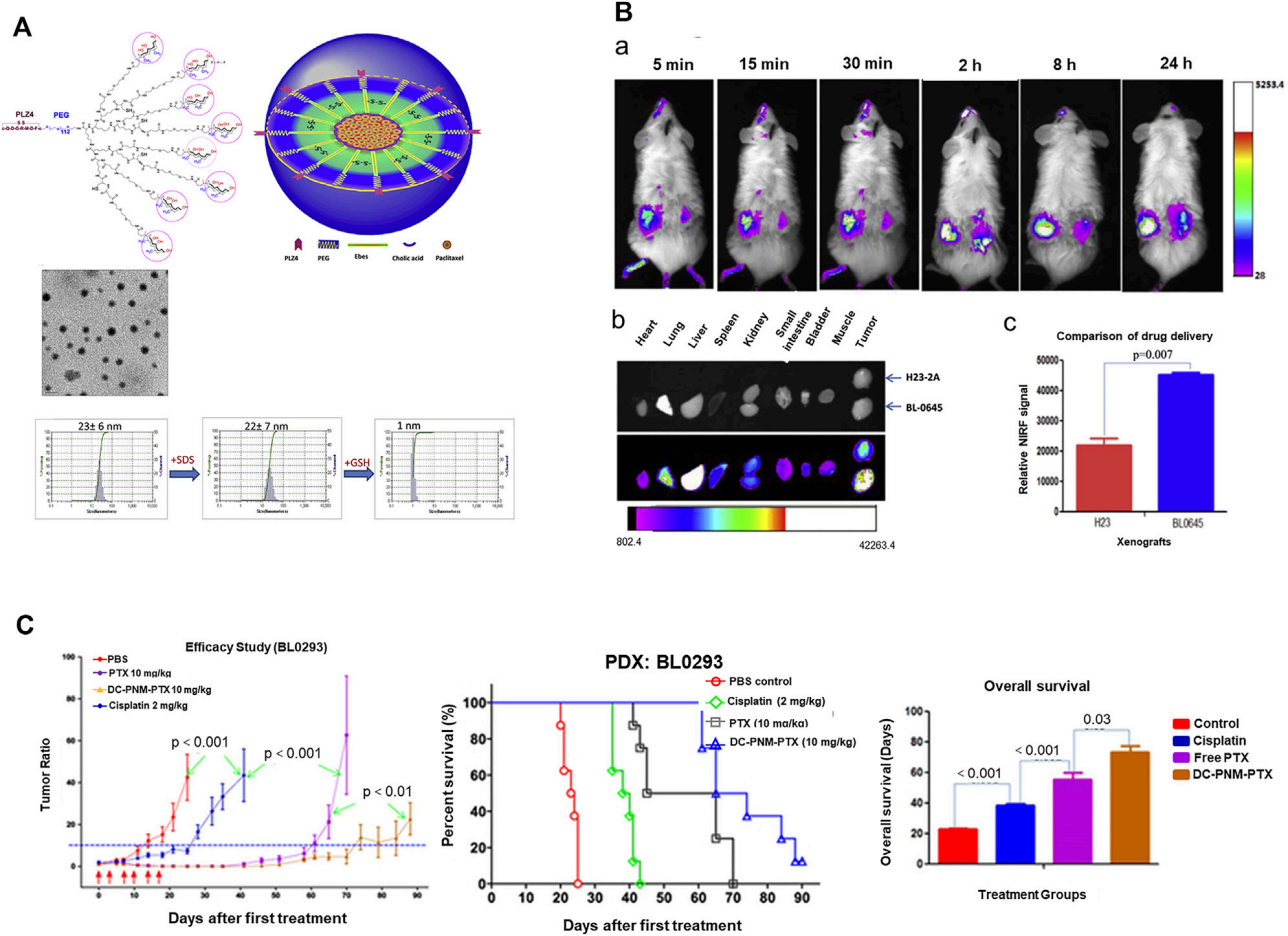
Reducing responsive nanogel for targeted drug delivery and enhancement of the drug curative effect. **(A)** Schematic diagram and characterization of PLZ4-telodendrimer and DC-PNM. **(B)** Delivery of DC-PNM and distribution of drugs in various organs *in vivo.* Reproduced with permission from (Pan et al., 2016).

The group of Lin further combined one end of polyethylene glycol with a cholic acid cluster and modified the other end with PLZ4. Daunorubicin (DNR) and paclitaxel (PTX) were loaded into the prepared micelles to form PLZ4-modified PLZ4-DNR/PTX nanoparticles with a particle size of about 23.2 ± 8.1 nm and their efficacy in dog bladder cancer examined *in vivo* and *in vitro*. As shown in Figure 7, compared with non-targeted nanomicelles, PLZ4-modified nanomicelles could deliver drugs to bladder cancer tissues more effectively and promote nanomicelle absorption from the bladder (Lin et al., 2012). *Pan* and co-workers developed bladder cancer-specific nanomicelles loaded with the chemotherapeutic drug paclitaxel (PTX). The micelles were gradually combined with polyethylene glycol, cholic acid and PLZ4. PTX and micelles were mixed and encased in the core, finally forming disulfide-crosslinked PLZ4 nanomicelles (DC-PNM-PTX). The drug loading rate of PTX was 25% and drug loading efficiency was >99%. The nanomicelles were spherical with a particle size of about 25 nm. Notably, upon intravenous administration *in vivo*, DC-PNM-PTX targeted bladder cancer but had no specificity for lung cancer in the same animal (Pan et al., 2016).

PLZ4 has therefore been identified as a target peptide for bladder cancer and effectively utilized as a modified vehicle for accurate delivery of drugs to achieve the maximum therapeutic effect.

#### 3.2.3 MPI-Modified Specific Targeted Therapeutic Nanocarriers for Bladder Cancer

Polybia mastoparans are novel mastoparan peptides purified from venom of the neotropical social wasp *Polybia paulista* (de Souza et al., 2009). Polybia mastoparan I (MPI), a member of the Polybia mastoparan family, is a tetradeca peptide with an amidated carbon terminal and three lysine residues in the fourth, fifth and 11th positions (Souza et al., 2005). The amino acid sequence of MPI is IDWKKLLDAAKQIL and its α-helical conformation promotes cell apoptosis and lysis. MPI selectively inhibits proliferation of bladder cancer cells with negligible toxicity to normal cells and has the advantages of high efficiency, specific targeting, biological safety, and excellent biological tolerance that are not observed with conventional chemotherapy drugs. However, the high molecular weight and high hydrophilicity of MPI cause insufficient bladder wall permeability, which limits the widespread promotion and application of this polypeptide (Wang et al., 2008; Orsolic, 2012; Li et al., 2019). Previous studies by Li et al. indicate that polymers with a higher degree of fluorination can improve the delivery efficiency of biomolecules. Recently, the delivery potential of F-PEI was examined as a new generation of transmucosal drug carriers through intravesical perfusion. After mixing, MPI polypeptides self-assembled with F-PEI to form MPI/F-PEI via the fluoride effect, hydrogen bonding and electrostatic interactions. The authors constructed a subcutaneous tumor model and administered free MPI, MMC, and MPI nanoparticles. Notably, MPI nanoparticles had an obvious inhibitory effect on tumor growth relative to free MPI and MMC. Based on these earlier studies, we infer that fluorinated polymers can be effectively used as transmucosal drug delivery vehicles with good application prospects for bladder cancer management, thus providing a new avenue for diversification of bladder cancer treatment in the future (Wang et al., 2008).

#### 3.2.4 c(RGDfK)-Modified Specific Targeted Therapeutic Nanocarriers for Bladder Cancer

The arginine-glycine-aspartic acid (RGD) peptide sequence with remarkable targeting ability specifically interacts with cells overexpressing αvβ3 integrin and plays a critical regulatory role in tumor growth, metastasis and angiogenesis (Zheng et al., 2020). High-affinity interactions between RGD peptides and tumor-associated integrins have led to their wide usage in construction of active targeting systems for anti-cancer drug delivery (Xiong et al., 2007; Zhan et al., 2010; Tian et al., 2011; Chen et al., 2015).

**FIGURE 8:**
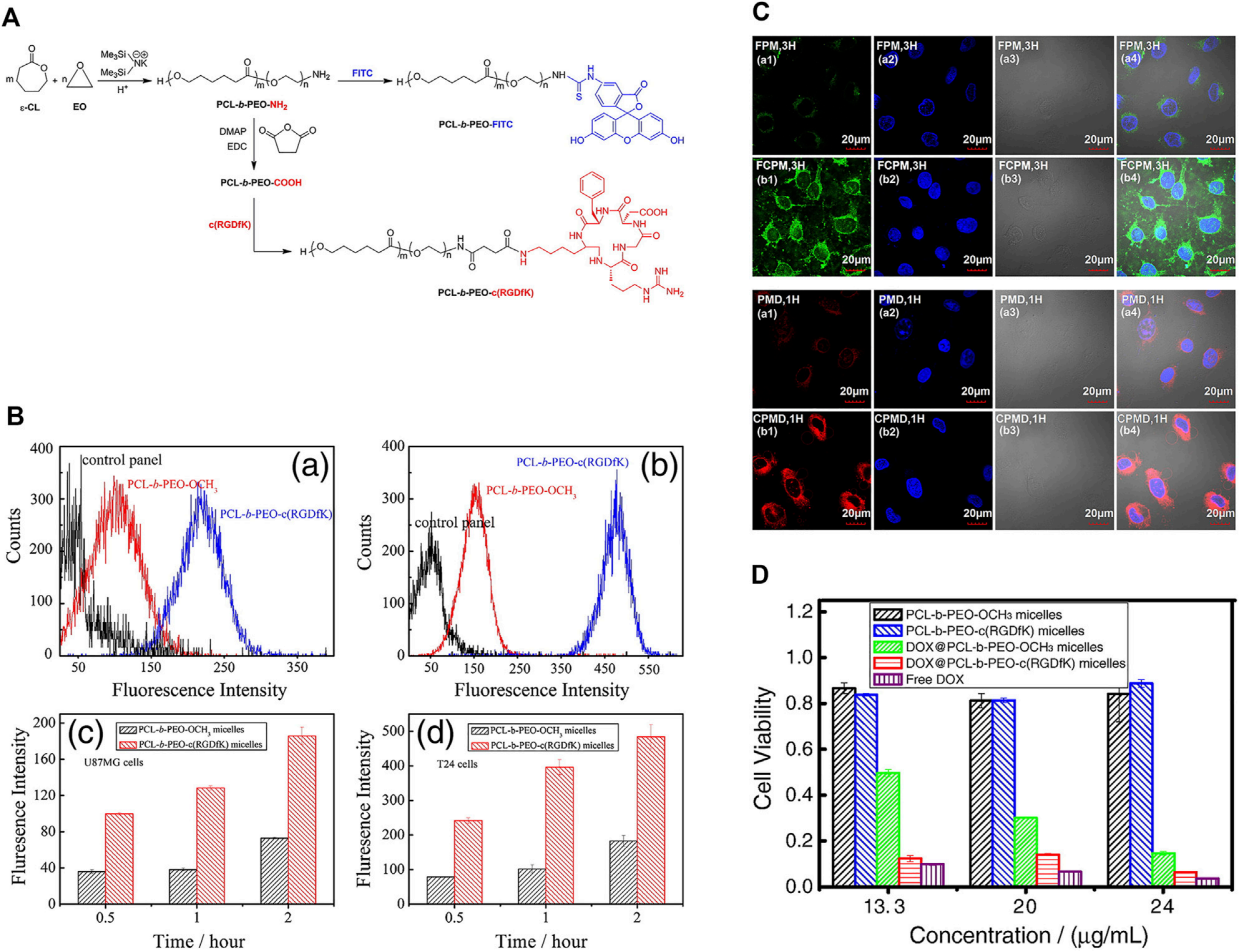
c (RGDfK) and FITC-modified PCL-b-PEO-NH_2_ for intravesical instillation therapy of bladder cancer **(A)** Synthetic routes of conjugation of c (RGDfK) and FITC to PCL-b-PEO-NH_2_. **(B)** Cellular uptake of U87MG (a, c) and T-24 cells treated with different nanogels for 2 h. **(C)** Confocal laser scanning microscopy images of T24 bladder cancer cells treated with FITC-labeled and Dox-loaded nanomicelles. **(D)** Viability of T24 bladder cancer cells treated with different drug-loaded nanomicelles. Reproduced with permission from (Zhou et al., 2013).

Zhou et al. (2013) developed a potential targeted drug delivery system for intravesical instilled chemotherapy for superficial bladder cancer. In this study, amphiphilic diblock copolymer polycaprolactone-b-polyethylene oxide (PCL-b-PEO), cyclic (arginine-glycine-aspartic acid-D-phenylalanine-lysine) (c (RGDfK)) and FITC were conjugated through specific end groups of the hydrophilic block and assembled into micelles. Interactions between micelles and various model cells were analyzed using confocal laser scanning microscopy and flow cytometry. The c (RGDfK) on the micelle surface was confirmed via ^1^H-NMR analysis and affinity for human glioblastoma-astrocytoma cells (U87MG). The *in vitro* cytotoxicity assay was used to evaluate the viability of bladder cancer cells (T-24) after incubation with doxorubicin (DOX) polymer micelles. The results showed strong affinity of c (RGDfK)-modified micelles for T-24 cells and significant inhibitory effect on cell proliferation when loaded with doxorubicin drugs, supporting a significant effect of c (RGDfK) on bladder cancer. The c (RGDfK)-modified micelles assembled by PCL-b-PEO diblock copolymer developed in this study displayed remarkable potential as a nano-drug system for superficial bladder cancer bladder perfusion chemotherapy (Figure 8).

#### 3.2.5 Protein-Modified Specific Targeted Therapeutic Nanocarriers for Bladder Cancer

The epidermal growth factor receptor (EGFR) is a focus of increasing research attention in the field of cancer treatment. EGFR is a member of the tyrosine kinase receptor family and its abnormal expression is associated with excessive cell proliferation. In addition, EGFR is reported to promote angiogenesis in tumors and inhibit growth and development of tumor cells (Yewale et al., 2013). EGFR is also overexpressed in bladder cancer and shown to be related to tumor stage, progression and clinical results. Two main EGFR inhibitors have been identified, specifically, monoclonal antibodies (Sartore-Bianchi et al., 2009) and small-molecule tyrosine kinase inhibitors (MAARTEN L. Janmaat, 2003). C225, a monoclonal antibody also known as cetuximab, mainly acts on the extracellular domain of EGFR. C225 competes with ligands for binding to EGFR, thereby blocking activation of the receptor and achieving treatment effects (Yao et al., 2019).

In terms of nanotherapy, Cho and co-workers synthesized multifunctional nanoclusters of upconversion nanoparticles (UCNP) and gold nanorod (AuNR) through PEGylation. The UCNP-AuNR multifunctional nanoclusters generated could be effectively applied to treat bladder cancer. Subsequently, these nanoclusters were further modified with antibodies to generate functionalized UCNP-AuNR with C225 antibody. In cell experiments, functionalized nanoclusters exerted obvious cytotoxic effects relative to those without antibody modifications. The use of monoclonal antibodies to modify nanocarriers is proposed to effectively reduce the dosage and maximize the therapeutic effects of drugs (Cho et al., 2019).

### 3.3 Hyaluronic Acid-Modified Specific Targeted Therapeutic Nanocarriers for Bladder Cancer

Owing to its multiple biological properties, such as high hydrophilicity and swelling capacity, hyaluronic acid (HA) is utilized in various drug carrier systems (Turcsanyi et al., 2020). HA is enriched in the extracellular matrix and secreted by cancer cells (Banerjee et al., 2016). In bladder cancer, tumor stroma and tumor cells can synthesize HA. CD44, a multifunctional cell surface transglycoprotein and a member of the cell adhesion molecule family overexpressed in various tumor cells, is involved in the growth, metastasis and apoptosis of several cancer cell types.

**FIGURE 9:**
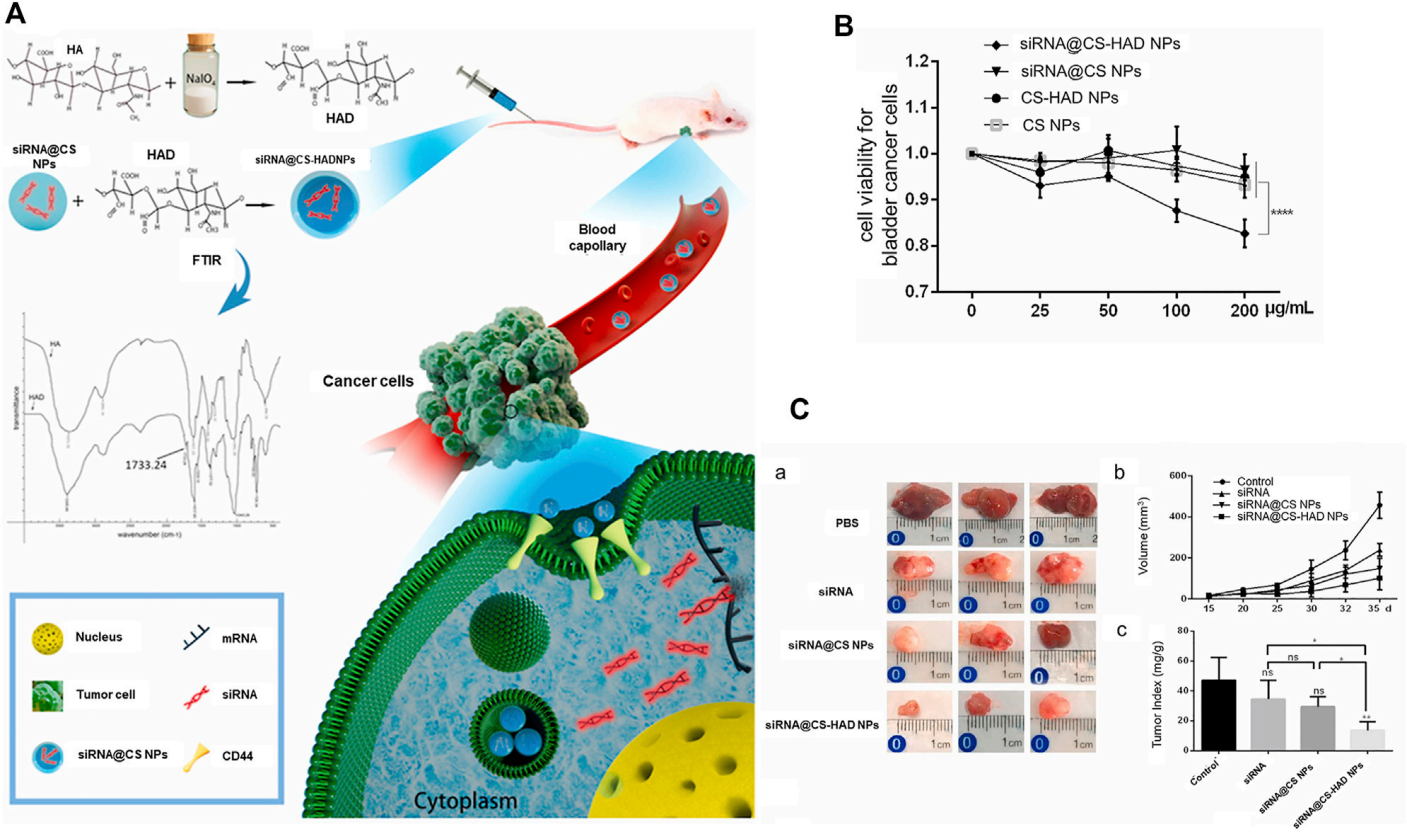
Hyaluronic acid-modified chitosan nanoparticles delivering targeted siRNA therapy for BC. **(A)** Schematic diagram of targeted delivery of siRNA for treatment of BC. **(B)** Viability of T24 BC cells treated with four types of nanoparticles for 96 h. **(C)** a: Images of bladder tumors after different treatments collected from sacrificed mice after the experiment. b: Regular measurements of changes in body weights of mice, c: Ratio of tumor weight to body weight. Reproduced with permission from (Liang et al., 2021).

Liang and co-workers confirmed markedly higher expression of CD44 in bladder cancer relative to normal bladder. CD44 was further identified as a specific receptor for HA. Combination of HA and CD44 could induce conformational changes and allow adaptor proteins or cytoskeleton elements to bind intracellular domains, thereby activating multiple signaling pathways for cell proliferation, adhesion and metastasis (Ponta et al., 2003; Zoller, 2011). The authors designed and screened siRNAs that could interfere with the Bcl2 gene and subsequently prepared CS-HAD nanoparticles targeting CD44 for siRNA delivery in an ethanol-water mixture. This system delivered siRNA to T24 bladder cancer cells through a ligand receptor-mediated targeting mechanism, ultimately interfering with expression of the apoptosis gene Bcl2. The siRNA@CS-HAD NP nano-system had a particle size of 100–200 nm, good stability, and strong siRNA encapsulation ability. The results of *in vivo* and *in vitro* experiments showed that siRNA@CS-HAD nanoparticles effectively suppressed bladder cancer growth without exerting significant biological toxicity. The delivery of cy3-siRNA promoted targeted gene silencing. *In vivo*, siRNA@CS-HAD NPs accumulated in BC tissues and exerted a strong inhibitory effect on target oncogenes and tumor growth. This novel nanosystem may therefore present an effective method for targeted treatment of BC with high CD44 expression (Liang et al., 2021) (Figure 9).

## 4 Conclusion and Future Perspectives

Bladder cancer is a global disease with high incidence worldwide. Treatment of bladder cancer generally entails surgical removal followed by intravesical instillation of chemotherapy into the bladder to prevent recurrence. However, conventional chemotherapeutic drugs are not selective for normal tissue cells and trigger numerous side-effects when frequently instilled. For example, instillation of conventional chemotherapeutics into the bladder is associated with chemical cystitis and hematuria, with the severity being significantly dependent on the dose and frequency of instillation. Moreover, the majority of adverse effects can be improved after termination of drug treatment. Due to the higher recurrence and progression rate of BC, patients require long-term and repeated intravesical instillation after surgery. However, frequent intravesical instillation of chemotherapy is associated with several problems, such as high cost and excessive family burden. In recent years, nanotechnology has been applied to resolve these issues and shown to be feasible and efficient for diagnosis and therapy of bladder cancer.

According to their characteristics, nanoparticles are divided into actively targeted and passively targeted therapeutic carriers. The fundamental principle of passive targeted therapy is the EPR effect while the active targeted drug delivery system uses specific ligands for attachment to vehicles or modification of the nanocarrier surface. Using the above mechanisms, nanoparticles can overcome the limitations of current traditional drug treatments for tumors, leading to significant improvement of therapeutic activity along with reduction of the side-effects of drugs. For instance, the nanoparticle surface induces significant changes in nanocarrier properties and interactions with the surrounding environment. At the same time, nanoparticles carry charges and non-specifically bind tumors through electrostatic interactions. As described above, positively charged nano-drug delivery carriers formed by chitosan bind non-specifically to the negatively charged inner wall of the bladder, leading to enhanced drug pairing. Adhesion to the bladder wall prolongs contact time with the tumor and enhances drug effects to an extent. Increasing attention has focused on tumor-specific targeted therapy and further research is ongoing.

Targeting ligands bound to the surface of nanoparticles can efficiently recognize receptors expressed on cancer cells, significantly improving the selectivity and killing effects of chemotherapeutics on bladder cancer cells. Targeting ligands generally include protein/peptide, hyaluronic acid, folic acid, and polysaccharides. Numerous *in vivo* and *in vitro* experiments on nanoparticles modified with targeting ligands and encapsulating chemotherapeutic drugs have confirmed that receptor-mediated endocytosis increases chemotherapeutic drug uptake by cancer cells. Targeted nanoparticle delivery carriers have several advantages over general nanocarriers. Firstly, these carriers are selective for bladder cancer cells, reducing their combination with normal bladder tissues and cells, and decreasing the side-effects caused by drug perfusion in the bladder. Secondly, drug-loaded nano-systems accumulate at tumor targets and release the drug, increasing therapeutic drug concentrations at the tumor tissue sites. Thirdly, specific ligand-receptor binding can extend the drug retention time in the bladder and prevent its excretion with urine. Fourthly, the release time and speed of the drug can be controlled to reduce or even avoid irritation to the bladder caused by multiple infusions of the drug.

Despite the numerous benefits of targeted nanoparticles in tumor treatment, several problems need to be resolved before clinical application, for example, between the experimental stage of nanomedicine and its transformation into a clinical agent, the technical problems of large-scale production of nanomedicines, and cost control during the manufacturing process. Moreover, immunotherapy has also made major clinical breakthroughs and it may be applied to bladder cancer in the future, which will greatly improve the therapeutic effect of bladder cancer (Zheng et al., 2021b; Feng et al., 2021; Sun et al., 2021).

In summary, although the efficacy of nanotherapy for bladder cancer remains to be established, its potential in targeted treatment and drug release provides a sound theoretical and practical basis for application in personalized treatment of bladder cancer.
